# Interaction of *Pseudostellaria heterophylla* with *Fusarium oxysporum* f.sp. *heterophylla* mediated by its root exudates in a consecutive monoculture system

**DOI:** 10.1038/srep08197

**Published:** 2015-02-03

**Authors:** Yongpo Zhao, Linkun Wu, Leixia Chu, Yanqiu Yang, Zhenfang Li, Saadia Azeem, Zhixing Zhang, Changxun Fang, Wenxiong Lin

**Affiliations:** 1 Fujian Provincial Key Laboratory of Agroecological Processing and Safety Monitoring (Fujian Agriculture and Forestry University), Fuzhou 35002, China; 2 College of Life Sciences, Fujian Agricultural and Forestry University, Fuzhou 35002, China

## Abstract

In this study, quantitative real-time PCR (qPCR) was used to determine the amount of *Fusarium oxysporum*, an important replant disease pathogen in *Pseudostellaria heterophylla* rhizospheric soil. Moreover, HPLC was used to identify phenolic acids in root exudates then it was further to explore the effects of the phenolic acid allelochemicals on the growth of *F. oxysporum* f.sp. *heterophylla*. The amount of *F. oxysporum* increased significantly in *P. heterophylla* rhizosphere soil under a consecutive replant system as monitored through qPCR analysis. Furthermore, the growth of *F. oxysporum* f.sp. *heterophylla* mycelium was enhanced by root exudates with a maximum increase of 23.8%. In addition, the number of spores increased to a maximum of 12.5-fold. Some phenolic acids promoted the growth of *F. oxysporum* f.sp. *heterophylla* mycelium and spore production. Our study revealed that phenolic acids in the root secretion of *P. heterophylla* increased long with its development, which was closely related to changes in rhizospheric microorganisms. The population of pathogenic microorganisms such as *F. oxysporum* in the rhizosphere soil of *P. heterophylla* also sharply increased. Our results on plant-microbe communication will help to better clarify the cause of problems associated with *P. heterophylla* under consecutive monoculture treatment.

P*seudostellaria heterophylla* belongs to the family *Caryophyllaceae*, and is a medicinal plant for over 3000 years mainly produced in a geo-authentic production zone, Zherong city, Fujian Province, southeast China. *P. heterophylla* root tubers are commonly used to treat chronic diseases associated with the lung and as a spleen tonic^1^. However, this plant suffers from serious consecutive monoculture problems. The monocultured plants are prone to severe diseases such as the outbreaks of severe fungal disease, resulting in reduced tuberous products. Thus, it is important to characterize the underlying mechanism to cause the problems with *P. heterophylla* under consecutive monoculture.

Three major causes have been described for consecutive monoculture problems in previous studies: imbalance of soil nutrients, autotoxicity of root exudates and shifts in the microbial community^2^. The autotoxicity issue attracted considerable attention^3^,^4^,^5^,^6^. This issue is not only observed in consecutively monocultured *P. heterophylla*, but also in many other greenhouse crops, fruits, forages, horticultural and medicinal plants. Several groups of chemicals have been implicated for autotoxicity, including terpenoids, phenolics, steroids, alkaloids and cyanogenic glycosides^7^,^8^,^9^,^10^.

However, many other studies suggested that the autotoxicity of root exudates could shape rhizosphere microbiology by deterring or attracting specific microbial species^11^,^12^. The root-associated microbial community plays a key role in the soil ecosystem and influences many biochemical processes in the soil and therefore plant fitness. Autotoxicity as a result of root exudates and the microbes affected by the exudates in the rhizosphere is detrimental to plant health^13^,^14^. The *p*-coumaric acid in cucumber root exudates significantly affects soil microbial communities in the rhizosphere and promoted the growth of soil-borne pathogens^15^. Qu and Wang^16^ artificially applied phenol 2,4-di-tert-butylphenol (PEDT) and vanillic acid (VA), two autotoxins from soybean root exudates, to the soil and found a significant impact of VA on microbial communities and an important role to cause soybean replant problems.

Based on our field observations, severe fungal diseases, especially root rot and Fusarium wilt, are commonly found in the *P. heterophylla* monoculture system. We hypothesize that some allelopathic compounds may have an indirect detrimental impact on plants, and predispose roots to infection by pathogenic fungi through certain biochemical and physiological interactions. Additionally, root allelochemicals in the rhizosphere have an ecological role in plant-microbe-soil interactions^17^. Allelopathic interactions associated with consecutive monoculture problems (replant disease) and rhizospheric microbial communities, as well as the ecological effects of root exudates deserve more detailed studies.

*Fusarium oxysporium* has numerous specialized forms, and is considered one of the most important fungal pathogens of crops that causes root rot and wilt disease^18^. It is also an important soil fungus in the rhizosphere of *P. heterophylla*. Previous studies^19^ using the T-RFLP (Terminal restriction fragment length polymorphism) method demonstrated that consecutive monoculture altered the rhizospheric microbial composition, with fewer beneficial microorganisms and more pathogenic microorganisms, suggesting that the continual monoculture had a negative effect on *P. heterophylla* growth and development. Nevertheless, how the specific pathogenic microorganism such as *F. oxysporum* varies in consecutive monoculture system and how the root exudates affect the soil-borne fungi under in vitro conditions still remain unknown. Therefore, the aim of the study is to quantify the population of the specific pathogen in rhizosphere soils of *P. heterophylla* with different years of monoculture using qPCR, and consequently to further assess the stimulatory effect of allelochemicals produced by host on the growth of soil-borne fungi and its relationships with consecutive monoculture problems. This paper provides a new insight into understanding the chemoecological process of host–pathogen interactions in rhizosphere, and attempts to explain the increased incidence of root rot and Fusarium wilt disease in the monocultured *P. heterophylla*.

## Results

### Dynamic of *F. oxysporum* in the rhizosphere soils of *P. heterophylla*

We first developed a standard curve y = -3.5919x + 12.753 (R^2^ = 0.993) (Amplification efficiency E = 10^−1/slope^ = 1.898) for our qPCR analysis. The result of qPCR analysis showed a significant increase in the amount of pathogenic *F. oxysporum* in the rhizosphere of *P. heterophylla* as the monoculture years increased (Table 1). This is consistent with the observation that the soil-borne disease becomes more severe with the increasing years of monoculture.

### The effects of root exudates on *F. oxysporum* f.sp. *heterophylla*

**Figure 1:**
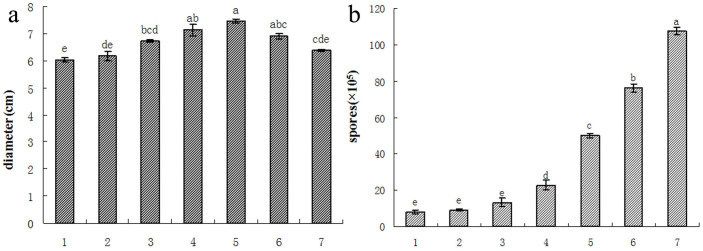
The effects of different concentrations of root secretion on the growth of *F. oxysporum* f.sp. *heterophylla*. (a) The effects on the hyphal growth and (b) the effects on the spore production. 1: control, 2: 1.25 μg/mL, 3: 2.5 μg/mL, 4: 5 μg/mL, 5: 10 μg/mL, 6: 20 μg/mL, 7: 30 μg/mL

Compared with the control, a significant increase in the growth of *F. oxysporum* f.sp. *heterophylla* mycelium was observed after a treatment with root exudates at concentrations ranging from 1.25 to 10 μg/mL (Figure 1a). The mycelium growth was significantly different in the presence of different concentrations of root exudates. Moreover, the mycelium growth plateaued at 10 μg/mL with a maximum stimulatory rate of 23.8%. Compared with the control, the spore production capacity was also increased significantly as the concentrations of root exudates were increased from 0 to 30 μg/mL and the number of spores increased to a maximum of 12.5-fold (Figure 1b).

### Quantification of phenolic acids in root exudates and their effects on *F. oxysporum* f.sp. *heterophylla*

**Figure 2:**
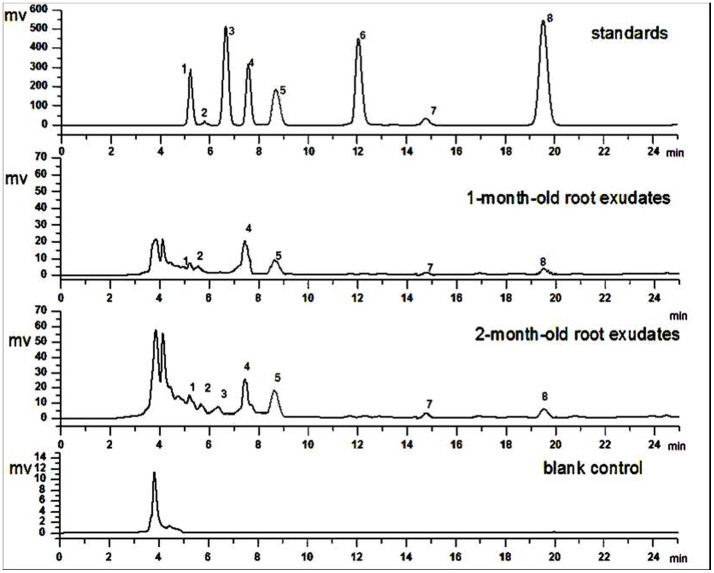
The contents of phenolic acids in the root secretions of *P. heterophylla* as analyzed with HPLC. 1: *p*-hydroxybenzoic acid, the retention time was 5.196 min; 2: vanillic acid, the retention time was 5.771 min; 3: syringic acid, the retention time was 6.386 min; 4: vanillin, the retention time was 7.677 min; 5: ferulic acid, the retention time was 8.433 min; 6: benzoic acid, the retention time was 12.318 min; 7: salicylic acid, retention time was 14.56 min; 8: cinnamic acid, the retention time was 19.624 min.

**Figure 3:**
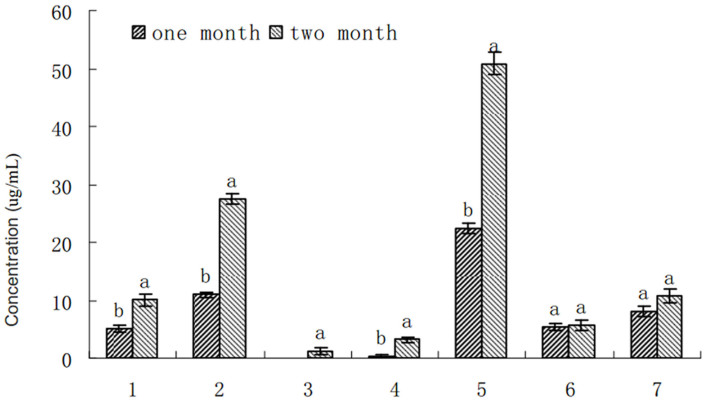
The quantitative analysis of phenolic acids in root exudates of *P. heterophylla.* 1: *p*-hydroxybenzoic acid, 2: vanillic acid, 3: syringic acid, 4: vanillin, 5: ferulic acid, 6: salicylic acid, 7: cinnamic acid.

Several organic acids including *p*-hydroxybenzoic acid, vanillic acid, vanillin, ferulic acid, salicylic acid and cinnamic acid were identified in root exudates of *P. heterophylla* grown for either 1 or 2 months, except syringic acid detected only at 2 months (Figure 2). Quantitative analysis with HPLC showed ferulic acid concentrations of 22.4 μg/mL MS medium and 50.9 μg/mL MS medium in root exudates of *P. heterophylla* cultivated for 1 and 2 months, respectively (Figure 3). This was the highest level of all the phenolic acids tested in this system, the next was vanillic acid, and *p*-hydroxybenzoic acid thereafter. Therefore, ferulic acid, vanillic acid and *p*-hydroxybenzoic acid are the main allelochemicals in the root exudates of *P. heterophylla*, especially grown for 2 months.

**Figure 4:**
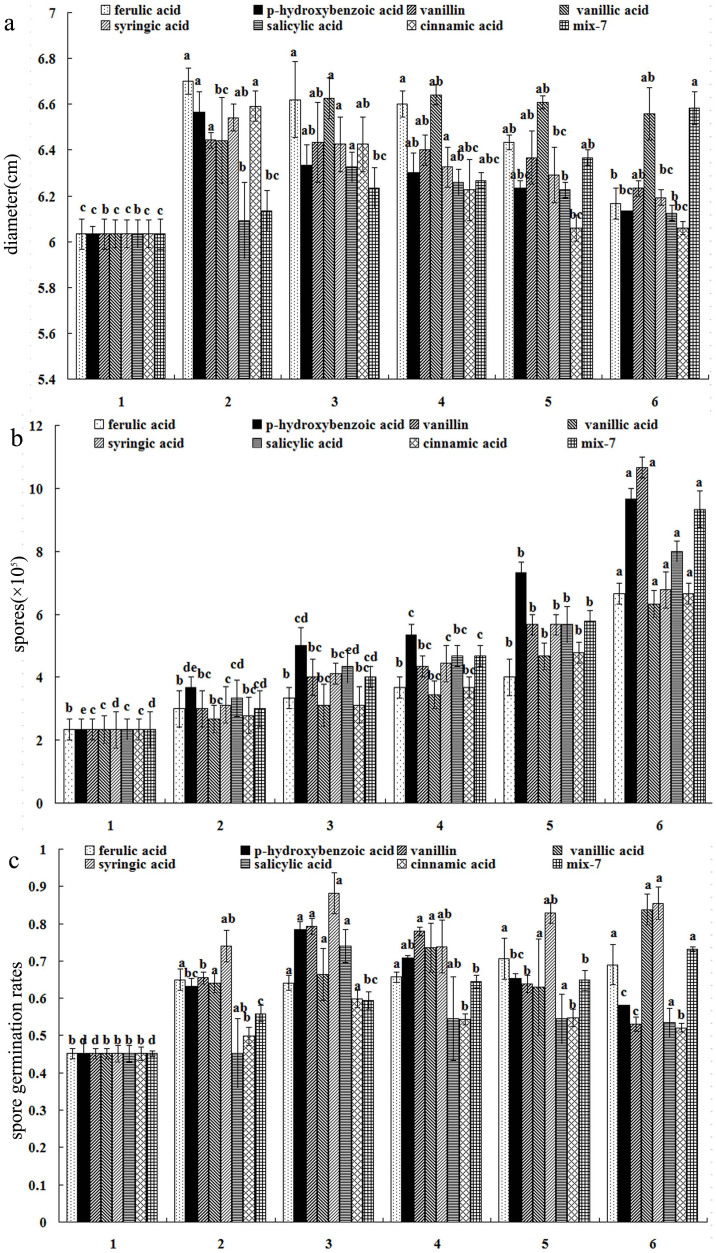
The effects of phenolic acids on the growth of *F. oxysporum* f.sp. *heterophylla*. (a) The effects on the hyphal growth, (b) the effects on the spore production and (c) the effects on the spore germination*.* 1: control, 2: 0.67 μg/mL, 3: 2 μg/mL, 4: 6 μg/mL, 5: 18 μg/mL, 6: 54 μg/mL.

The mycelial growth patterns of *F. oxysporum* f.sp. *heterophylla* treated with a single phenolic acid and their mixture indicated that these phenolic acids at concentrations below 0.67 μg/mL could promote fungal mycelium growth (Figure 4a). Among the compounds, ferulic acid showed the highest stimulatory rate on the mycelium growth (11.1%). For most of single phenolic acid (i.e. ferulic acid, *p*-hydroxybenzoic acid, syringic acid and cinnamic acid), the growth rate reached a plateau at 0.67 μg/mL concentration. Therefore, a single phenolic acid may promote the mycelium growth of *F. oxysporum* f.sp. *heterophylla* within a specific concentration range. This result is consistent with the effect of total root exudates on the mycelium growth of *F. oxysporum* f.sp. *heterophylla* as observed above. However, for phenolic acids mixture, the promoting rate on mycelium growth increased as the concentration increased, implying that the synergistic effects of phenolic acids might be involved in the mixture.

Phenolic acids showed a significant dose-response effect on the capacity of *F. oxysporum* f.sp. *heterophylla* spore production at concentrations from 0 to 54 μg/mL (Figure 4b). Therefore, all phenolic acids enhanced the spore production of *F. oxysporum* f.sp. *heterophylla* at a range of concentrations. Vanillin showed the greatest enhancement (357%), followed by *p*-hydroxybenzoic acid (314%) and mix-7 (300%).

Treated by phenolic acids, the spore germination rate of *F. oxysporum* f.sp. *heterophylla* increased dramatically as compared with control (Figure 4c). For the single phenolic acid, most of them reached a plateau at 2 μg/mL concentration. However, the promoting rate of phenolic acids mixture on spore germination increased as the concentration increased, indicating the similarity to the effects of phenolic acids mixture on mycelial growth. Therefore, increased growth of the pathogenic fungi, *F. oxysporum* was closely related to the stimulation of phenolic acids in root exudates from the monocultured medicinal plants in the consecutive cropping system.

### Utilization of three key phenolic acids by *F. oxysporum* f.sp. *heterophylla*

Based on the previous results, standard curves for ferulic acid, *p*-hydroxybenzoic acid and vanillin were established using HPLC to detect the utilization of *F. oxysporum* f.sp. *heterophylla* on these three key phenolic acids. We then developed a standard curve equation for selected three compounds: *p*-hydroxybenzoic acid, y = 4471.4x + 8343.3 (R^2^ = 0.9995); vanillin, y = 13050x – 14191 (R^2^ = 0.9991); ferulic acid, y = 12194x + 38266 (R^2^ = 0.9998).

**Figure 5:**
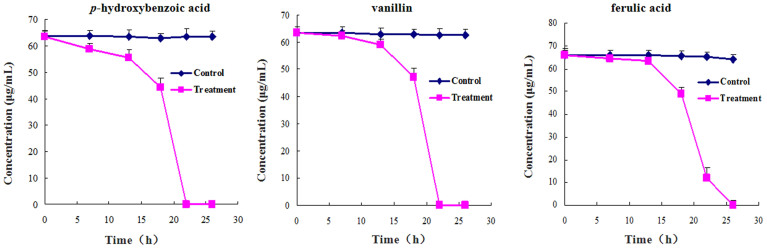
Utilization of three key phenolic acids by *F. oxysporum* f.sp. *heterophylla* under liquid fermentation.

We used HPLC analysis to monitor the amount of the three phenolic acids in the fermentation media of *F. oxysporum* f.sp. *heterophylla*. We observed different patterns in the usage of the three compounds by *F. oxysporum* f.sp. *heterophylla* as compared to the control. After 22 h, *p*-hydroxybenzoic acid and vanillin were completely consumed and after 26 h ferulic acid was completely consumed in the fermentation media (Figure 5).

## Discussion

Soil sickness or replanting disease, is a special allelopathy phenomenon, known as autotoxicity or a consecutive monoculture problem. It refers to a chemoecological phenomenon of plant dysplasia, serious diseases and significant decline in yield and quality caused by consecutively planting the same plants in the same land for many years. Many crops suffer from the soil sickness in modern cropping system^2^,^16^,^20^. The present study suggests that soil sickness is a typical negative plant-soil feedback with a reduction in crop yield and a prevalence of soil borne diseases. Various factors such as soil nutrient imbalance, autotoxins generation and/or change of soil microbial community structure are considered as fundamental factors on the soil sickness. These factors interact in a complex net of feedback, not split into a lonely individual. This study demonstrated that the replanting disease or autotoxicity of *P. heterophylla* results from its interactions with specific pathogenic organisms mediated by allelochemicals existing in its root exudates from the consecutively monocultured medicinal plants in the cropping sequence. In our previous study, we used the T-RFLP (Terminal restriction fragment length polymorphism) method to study the rhizospheric microbial composition under consecutive monoculture system of *P. heterophylla* and we found that the poor growth of *P. heterophylla* under consecutive monoculture system resulted from an altered microbial community (with fewer beneficial microorganisms and more pathogenic microorganisms)^19^. Accordingly, based on the highest separation frequency and the result of the pathogenic infection and its verification, qPCR approach was employed to detect the size of the specific pathogenic population in situ rhizosphere soil under a consecutive monoculture system for different years. The result showed that the population of *F. oxysporum* in the rhizosphere of *P. heterophylla* significantly increased under a monoculture system, to a level fivefold higher than that of the control (Table 1), which further confirmed our previous hypothesis that the consecutive monoculture problems or replanting diseases resulted from the proliferation of some pathogenic fungi^16^,^19^,^21^.

The previous studies suggested that accumulation of plant root exudates in the rhizosphere after monoculture could cause rapid pathogen proliferation in replanted soil, which plays a crucial role in soil quality, crop health and yield^22^. In our study, in order to overcome the interference of soil microbes, we determined the allelochemicals in root exudates from tissue culture plantlets of *P. heterophylla* using HPLC method under sterile condition in laboratory, it was found that the root exudates contain rich phenolic acids, such as *p*-hydroxybenzoic acid, vanillic acid, vanillin, ferulic acid, salicylic acid and cinnamic acid, which were identified in the exudates from the roots of the tissue culture plantlets grown for either 1 or 2 months, whereas syringic acid was only present in root exudates from tissue culture plantlets of *P. heterophylla* grown for 2 months under sterile condition in this study. Quantitative analysis with HPLC showed that ferulic acid, vanillic acid and *p*-hydroxybenzoic acid were the main allelochemicals in terms of their concentrations in the root exudates of *P. heterophylla*, especially grown for 2 months under sterile condition. Further analysis in sequent experiments confirmed that the increased amount of *F. oxysporum* in situ rhizosphere soil of *P. heterophylla* was related to the stimulation of phenolic acids in root exudates under the monoculture system, indicating that the growth of *F. oxysporum* f.sp. *heterophylla* and the germination of the microbial spores were promoted dramatically by single identified phenolic acid and their mixture in the same ratio as the results of HPLC analysis in root exudates of *P. heterophylla* with treated dosage increasing. Especially, we found that the mixture of the phenolic acids showed increasing stimulatory effect on hyphal growth, spore production and germination of *F. oxysporum* f.sp. *heterophylla* as the concentration increased, which was contributed to interaction of the phenolic acids including additive, antagonistic and synergistic effects^23^. It is therefore suggested that using the mixture of phenolic acids in the same ratio as the results of HPLC analysis to bioassay is the better way closer to the real situation, however using single phenolic acid will overestimate its ecological effect on the target organisms.

Overall, our results explain the reason in part why the soil environment was prone to deterioration in consecutive monoculture system, which gets access to further understanding the mechanism of the increased incidence of root rot and Fusarium wilt disease in consecutively monocultured *P. heterophylla* in the cropping sequence, including that how the phenolic acids play important roles in interacting with the formae speciales *F. oxysporum*, and predisposing roots to be infected by pathogenic fungi and how the outbreak of pathogenic microorganisms influences the microbial community in the rhizosphere of *P. heterophylla*, subsequently leads to serious consecutive monoculture problems. It is therefore suggested that a comprehensive understanding of the mechanisms that governs selection and activity of the microbial communities by plant roots will provide new opportunities to improve crop production.

### Conclusions

The population of *F. oxysporum* in the rhizosphere of *P. heterophylla* increased with the increasing years of monoculture. Phenolic exudates from monocultured *P. heterophylla* significantly enhanced mycelium growth, spore production and germination of *F. oxysporum* f.sp. *heterophylla*. In short, replant disease of *P. heterophylla* mainly results from the increased proliferation of pathogenic *F. oxysporum* in the rhizosphere soil of *P. heterophylla* stimulated by its root exudates (especially phenolic compounds) in a consecutive monoculture system.

## Methods

### Plant and soil collection

In this study, the trial field located at the *Pseudostellaria heterophylla* Demonstration District of Agroecological Institute, Fujian Agriculture and Forestry University, Zherong (119.89479E,27.23856N), Fujian, is known as a geo-authentic production zone, i.e., an optimal production area for *P. heterophylla*. The rhizosphere soil samples of *P. heterophylla* were collected from both newly planted and replanted fields at the harvesting time, and the adjacent uncultivated field soil sample was collected as a control. A total of five random plots (15 m^2^) for each rhizosphere soil sample were used in our trial tests.

### Fungal Pathogen Isolation

*Fusarium oxysporum* f.sp. *heterophylla* was isolated from infected *P. heterophylla* plants grown in the *Pseudostellaria heterophylla* Demonstration District, the Agroecological Institute, Fujian Agriculture and Forestry University. The separation frequency was 88.5%, and it only infected *P. heterophylla* plants but was not able to infect the seedlings of other crops (i.e. corn, soybean, peanut and rice). We isolated the pathogenic fungus from the infected parts of *P. heterophylla* plants again and verified it to be exactly the same as the original strain (*Fusarium oxysporum*) based on morphological observation, infection characteristic and ITS sequence analysis. It has been preserved in the Agroecological Institute, Fujian Agriculture and Forestry University, P.R. China.

### Design of specific primers and PCR specificity analysis

The sequencing information of the pathogenic strains was searched using BLAST and homologous comparison. Specific primers YD1/YD2 (Table 2) were designed using Premier Primer 5.0 software and synthesized from Shanghai Sangon Co. Ltd (Shanghai, China). The amplified target fragment was 106 bp in length and named as Foh106.

DNA from *F. oxysporum, F. graminearum, F. solani, Aspergillus flavus, Bacillus amyloliquefaciens*, and the different-year monoculture soils was extracted using the cetyl trimethyl ammonium bromide (CTAB) method, and used to test the specificity of the primer sets YD1/YD2 and for subsequent PCR analysis^24^. PCR reactions were performed in 25 μL reaction mixtures that contain 0.5 μL of each primer, 1 μL DNA, 12.5 μL DNA mix and 10.5 μL ddH_2_O. Cycle conditions were 5 min at 95°C, 35 cycles of 30 s at 95°C, 30 s at 62.5°C and 20 s at 72°C, followed by 10 min of extension at 72°C and a final hold at 4°C. PCR products were analyzed on an agarose gel.

### Quantitative real-time PCR (qPCR) of *F. oxysporum* in the rhizosphere of *P. heterophylla* under different-year monoculture systems

The Foh106 fragment was gel-purified, cloned into the PeasyTM-T4 Zero Cloning Kit (Beijing TransGen Biotech Co., Ltd.), and sequenced. The sequenced DNA was reamplified using YD1/YD2 from the plasmid and purified using a TIAN pure Mini Plasmid Kit (TIANGEN BIOTECH (BEIJING) CO., LTD). The concentration of the target DNA (Foh106) was determined using a spectrophotometer (NanoDrop2000c, USA), and was then diluted to 5, 4, 3, 2, 1, 0.1, 0.01, and 0.001 ng/μL. qPCR was monitored on a Mastercycler ep realplex (Germany). Standard curve plotting and melting-curve analysis was performed following the qPCR amplification protocol^25^. The standard curve was created by plotting the target DNA concentration against the threshold cycle (Ct) value exported from the Mastercycler ep realplex. The primer sets YD1/YD2 were evaluated using the established standard curve and melting curves were determined using qPCR amplification in four replicates with a serial dilution of the target DNA template. qPCR reactions were performed in 15 μL reaction mixtures (7.5 μL 2 × SYBR Green PCR Master Mix, 0.25 μL YD1, 0.25 μL YD2, and 4 ng DNA made up to a final 15 μL with ddH_2_O). The parameters were as follows: 95°C for 5 min (denaturation and hot-start activation), followed by 40 cycles of 95°C for 10 s and 62.5°C for 20 s. After the qPCR ran, melting curve analysis was performed to verify the specificity of the amplified product under the following conditions: 95°C for 15 s, 60°C for 15 s, followed by an increase to 95°C over 10 min and a hold at 95°C for 15 s.

### Extraction of *P. heterophylla* root exudates and its influences on the mycelia growth, spore production and germination of *F. oxysporum* f.sp. *heterophylla*

Batches of uniform seedlings with healthy roots at the 4-leaf-stage were transferred into sterile robust seedling medium (MS medium supplemented with 1 mg/L 6-BA, 0.1 mg/L NAA, and 30 g/L sucrose). Each group included three replicates in 30 mL of MS medium and three tissue culture plantlets. The tissue culture plantlets were grown at 25°C under bacteria-free condition. The illumination time was from 6:00 to 18:00, the light intensity was 3.97 ± 0.15 × 10^3^ lux and the relative humidity was 50% in the growth chamber. All mediums were then collected after the tissue culture plantlets were incubated for one month and two months, respectively. Blank culture medium was used as a control. A total of 200 mL of methyl alcohol (chromatography) was added to each group mediums. After ultrasonic processing and incubating for several minutes, the extracting solution was centrifuged and concentrated using rotary evaporation at 40°C until the final volume was 5 mL, and then dried with N_2_, weighed and stored at −80°C. Thereafter, half of the exudates were dissolved with sterile distilled water to 100 μg/mL^26^,^27^, and then diluted to 30, 20, 10, 5, 2.5 and 1.25 μg/mL. *F. oxysporum* f.sp. *heterophylla* was inoculated onto a 9 cm diameter petri dish and cultivated at 28°C for 5–7 days. *F. oxysporum* f.sp. *heterophylla* growth diameter was measured when the control hyphal diameter covered two thirds of the culture dish.

Under sterile conditions, *F. oxysporum* f.sp. *heterophylla* spores were flooded with sterilized water and the conidia suspensions were filtered through three layers of gauze to separate conidia from mycelium^28^. The conidia suspensions were examined under a hemocytometer.

### Identification and quantification of phenolic acids in *P. heterophylla* root exudates by HPLC

Root exudates (100 μg/mL) of the monocultured *P. heterophylla* were filtered through a 0.22 µm filter prior to the analysis of the root exudation profiles. The analysis was conducted using an HPLC system (Shimadzu LC-20A, Japan) with an ODS - C18 column (Inertsil ODS-SP 4.6 mm * 250 mm, Japan). The root exudation profiles were determined under the following HPLC conditions, by which eight standard phenolic compounds (*p*-hydroxybenzoic acid, vanillic acid, vanillin, syringic acid, ferulic acid, benzoic acid, salicylic acid and cinnamic acid) could be completely separated. Under these conditions, the mobile phase consisted of methanol (A) and 1% phosphoric acid (B) with a gradient elution of 100–50% A plus 0–50% B at a rate of 0.8 mL/min for 12 min, 50% A plus 50% B at a rate of 0.8 mL/min for 20 min, 50–30% A plus 50–70% B at a rate of 0.8 mL/min for 30 min, 30% A plus 70% B at a rate of 0.8 mL/min for 32 min, and 30–50% A plus 70–50% B at a rate of 0.8 mL/min. The UV detector wavelength was set at 280 nm and the column temperature was maintained at 40°C. The standard compounds were chromatographed alone and in mixtures. Retention times for the standard compounds and the major peaks in the extracts were recorded. The phenolic compounds from each fraction were identified based on their retention times and the addition of standards to the samples^29^.

### The effects of phenolic compounds identified in *P. heterophylla* root exudates on the growth of *F. oxysporum* f.sp. *heterophylla*

Based on our preliminary study, seven standard compounds identified in root exudates were selected and made as mother solution, then diluted to 54, 18, 6, 2 and 0.67 μg/mL in each medium alone, at the same time, the mixtures with ferulic acid, *p*-hydroxybenzoic acid, vanillin, vanillic acid, syringic acid, salicylic acid and cinnamic acid were made in the same ratio as the results by HPLC analysis and diluted to the same concentrations as single phenolic acid dissolved in the basal medium (soil water extract agar medium^30^) with three replicates. The isolated *F. oxysporum* f.sp. *heterophylla* was inoculated onto 9 cm diameter petri dishes with the specific basal medium containing different concentrations of single phenolic acids and their mixtures, then incubated at 28°C for 5~7 days. The blank basal medium was used as control. The *F. oxysporum* f.sp. *heterophylla* growth diameter was measured when the control hyphal diameter covered two thirds of the culture dish. Under sterile conditions, *F. oxysporum* f.sp. *heterophylla* spores were flooded with sterilized water and the conidia suspensions were filtered through three layers of filter paper to separate conidia from mycelium. The conidia suspensions were examined under a hemocytometer. *F. oxysporum* f.sp. *heterophylla* conidia suspension of 10^5^/mL concentration was prepared to detect the effects of phenolic compounds on microconidia germination. Microconidia germination treated by phenolic compounds was incubated at 25°C for 7 h (germination rate of the control reached about 50%). After 7 h, 0.1% HgCl_2_ was added to all the experimental groups to stop spore germination. Then the spore germination rate was calculated. Each treatment had three replicates.

### Utilization of three key phenolic acids by *F. oxysporum* f.sp. *heterophylla*

A total of 100 μL of *F. oxysporum* f.sp. *heterophylla* (3 × 10^6^ microconidia/mL) and 64 μg/mL of each standard phenolic acid (ferulic acid, *p*-hydroxybenzoic acid and vanillin) were added to modified Czapek's medium (with sucrose removed). Basal medium was used as the control. *F. oxysporum* f.sp. *heterophylla* was then cultivated at 28°C with 150 rpm rotation. Each group comprised three replicates. The concentration of each phenolic acid was quantified in the liquid fermentation media using HPLC at 4–7 h intervals. Standard compounds of the three concerned phenolic acids (ferulic acid, *p*-hydroxybenzoic acid and vanillin) were calibrated with 5, 1, 0.5, and 0.1 μg/mL standard solutions for HPLC chromatography. The standard compounds were injected alone and in mixtures. Retention times for the standard compounds and the major peaks in the medium were recorded. The phenolic compounds from each fraction were identified based on their retention times and the addition of standards to the samples. The HPLC conditions were as following: The column was ODS - C18 (Inertsil ODS-SP 4.6 mm * 250 mm, Japan), with a mobile phase of methanol and 1% phosphoric acid aqueous solution (30: 70). The UV detector wavelength was set at 280 nm at a rate of 1 mL/min, and the column temperature was maintained at 40°C.

### Statistical Analysis

Analysis of variance was performed using DPS7.05. The differences in the means were determined by calculation of the least significant difference (LSD) at the 5% level.

## Author Contributions

W.X.L. designed the experiments, and Y.P.Z., Z.F.L. and L.X.C. executed the experiments and wrote the manuscript. W.X.L. improved the manuscript. Y.Q.Y., L.K.W., S.A., Z.X.Z. and C.X.F. helped to polish the language. All authors reviewed the manuscript.

## Supplementary Material

Supplementary Information Dataset 1

## Figures and Tables

**Table 1:** The content of *F. oxysporum* in *P. heterophylla* rhizosphere soils after different years of monoculture

Samples	Content (10^5^ cells/g fresh soil)
control soil without any crop planted	0.55 ± 0.040c
newly planted soil	1.9 ± 0.069b
replanted soil	2.7 ± 0.083a

**Table 2:** DNA sequences of the primer pairs used in this study

Oligonucleotide	Sequence
ITS1	5′CTTGGTCATTTAGAGGA AGTAA3′
ITS4	5′TCCTCCGCTTATTGATATGC 3′
YD1	5′GCAGCGAGACCGCCACTAGATTT3′
YD2	5′ TGCCTGTTCGAGCGTCATTTCA 3′
