# Supplementary material for: Interaction of Pseudostellaria heterophylla with Fusarium oxysporum f.sp. heterophylla mediated by its root exudates in a consecutive monoculture system
Source: Sci Rep. 2015 Feb 3;5:8197. doi: 10.1038/srep08197 (PMC4314652; doi:10.1038/srep08197)
Supplement: Supplementary Information — Dataset 1 [file srep08197-s1.pdf]

Title

Interaction of *Pseudostellaria heterophylla* with *Fusarium oxysporum* f.sp. *heterophylla* mediated by its root exudates in a consecutive monoculture system

Authors and e-mail address

Yongpo Zhao [1,2], Linkun Wu[1,2], Leixia Chu[1,2], Yanqiu Yang [1,2], Zhenfang Li [1], Saadia Azeem[1,2], Zhixing Zhang [1,2], Changxun Fang [1,2], Wenxiong Lin [1,2*]

*Corresponding author

Affiliations

1. Fujian Provincial Key Laboratory of Agroecological Processing and Safety Monitoring (Fujian Agriculture and Forestry University), Fuzhou 35002, China

2. College of Life Sciences, Fujian Agricultural and Forestry University, Fuzhou 35002, China

Full address for correspondence

*Corresponding author: Wenxiong Lin, Agroecological Institute, Fujian Agriculture and Forestry University, Fuzhou 35002, Fujian, People's Republic of China.

Phone +86-591-83737535 **Fax** +86-591-83769440

E-mail address 30403006@fafu.edu.cn

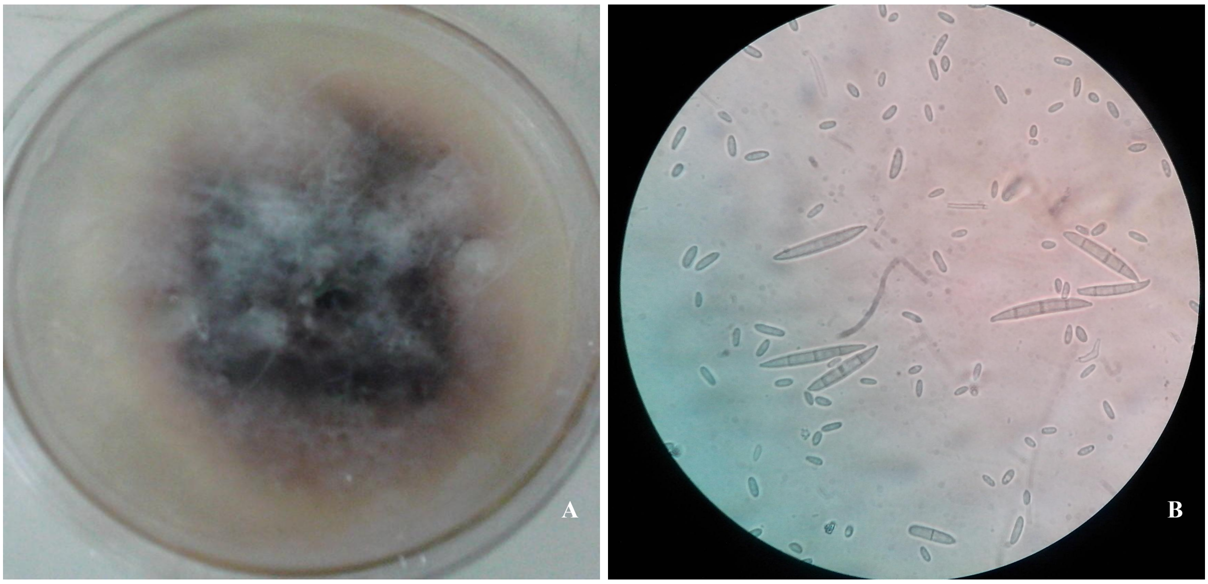

Figure S1. The morphologic observation of hypha and spores of *F. oxysporum* f. sp. *heterophylla*（400×）

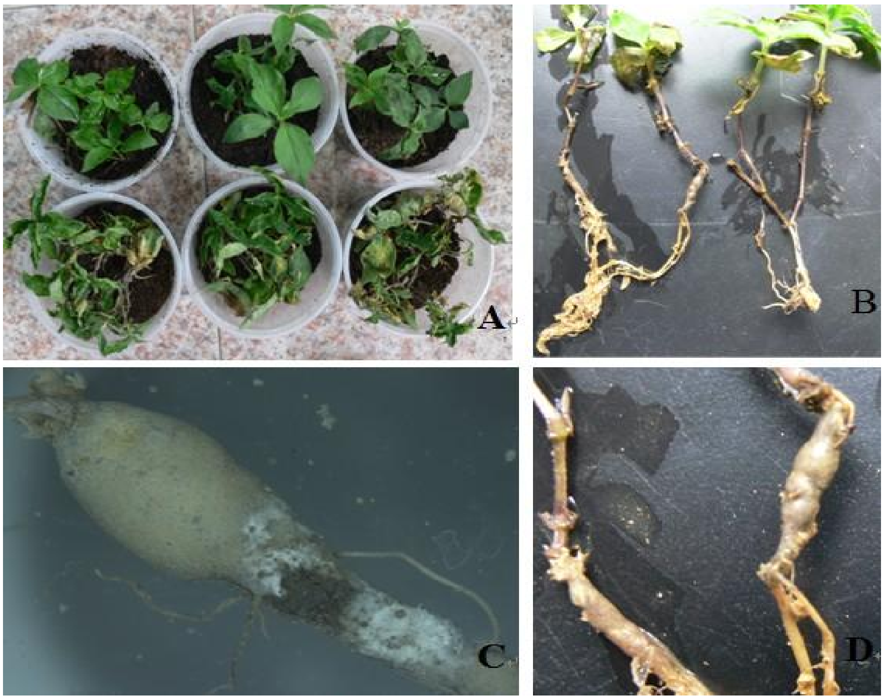

Figure S2. The pathogenicity test of *F. oxysporum* f. sp. *heterophylla*

A. The first line was as control, the second line was inoculated by *Fusarium* strains; B, C, D. *Pseudostellaria heterophylla* stem and root after *Fusarium* strains infection

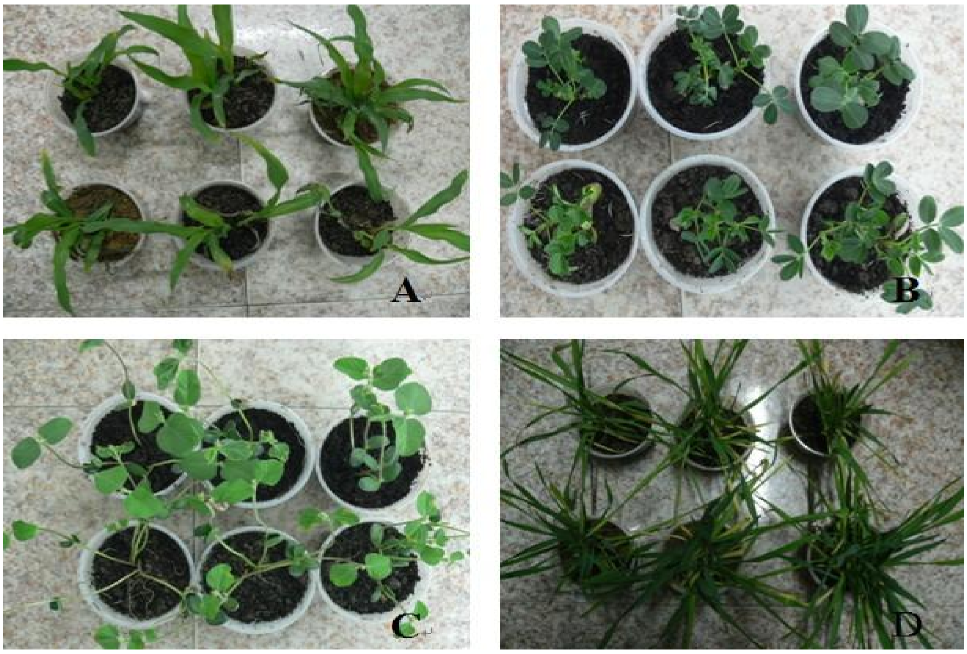

Figure S3. The growth of the tested materials infected by *F. oxysporum* f. sp. *heterophylla*
The first line was as control, the second line was inoculated by *Fusarium* strains; A. maize; B. peanut; C. soybean; D. rice.

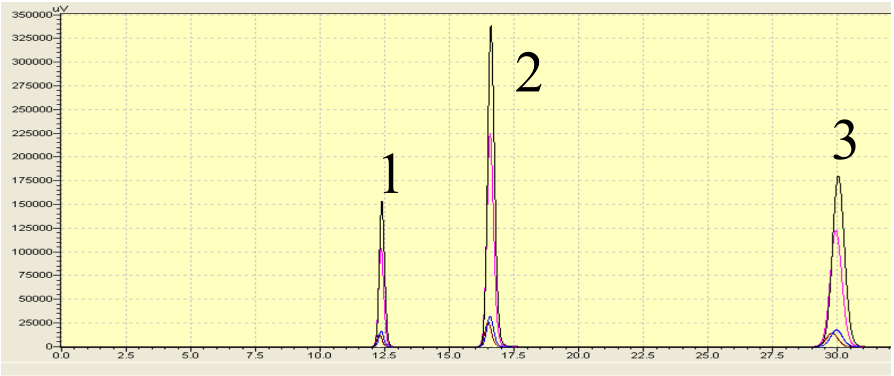

Figure S4. The standard curve of *p*-hydroxybenzoic acid, vanillin and ferulic acid.

1: *p*-hydroxybenzoic acid, the retention time was 12.8min; 2: vanillin, the retention time was 17.3min; 3: ferulic acid, the retention time was 31.5min.
